# Supplementary material for: Protective Immunity of COVID-19 Vaccination with ChAdOx1 nCoV-19 Following Previous SARS-CoV-2 Infection: A Humoral and Cellular Investigation
Source: Viruses. 2022 Aug 30;14(9):1916. doi: 10.3390/v14091916 (PMC9504152; doi:10.3390/v14091916)
Supplement: Supplementary file 1 [file viruses-14-01916-s001.zip › viruses-1864683-supplementary.pdf]

Supplementary Materials

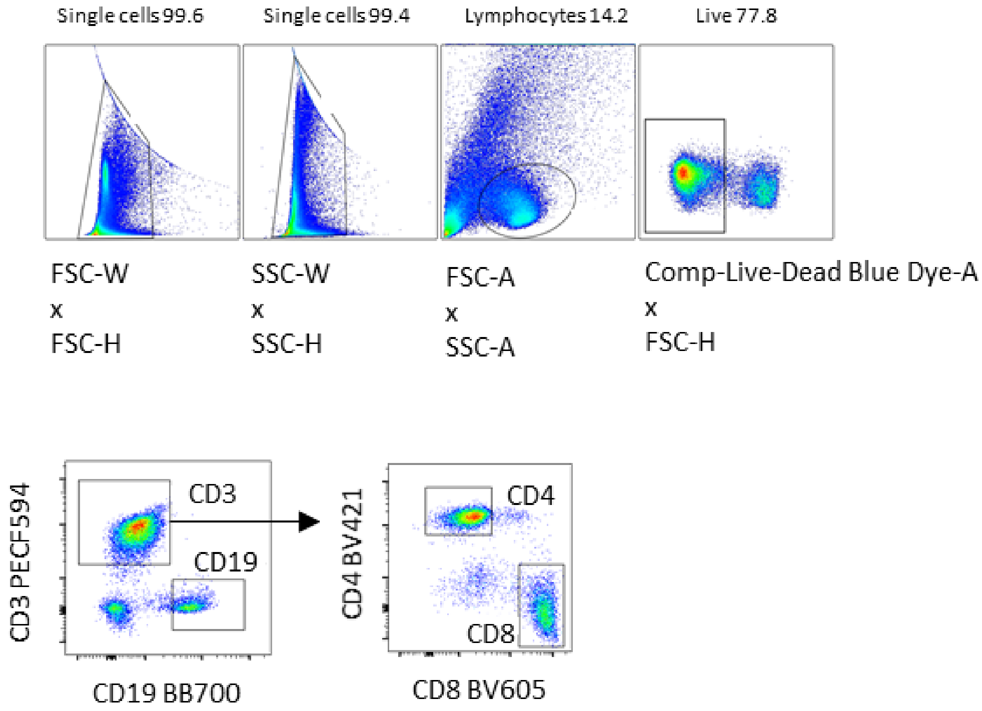

Supplementary Figure S1. Immunophenotype gating strategy for B and T memory cells.

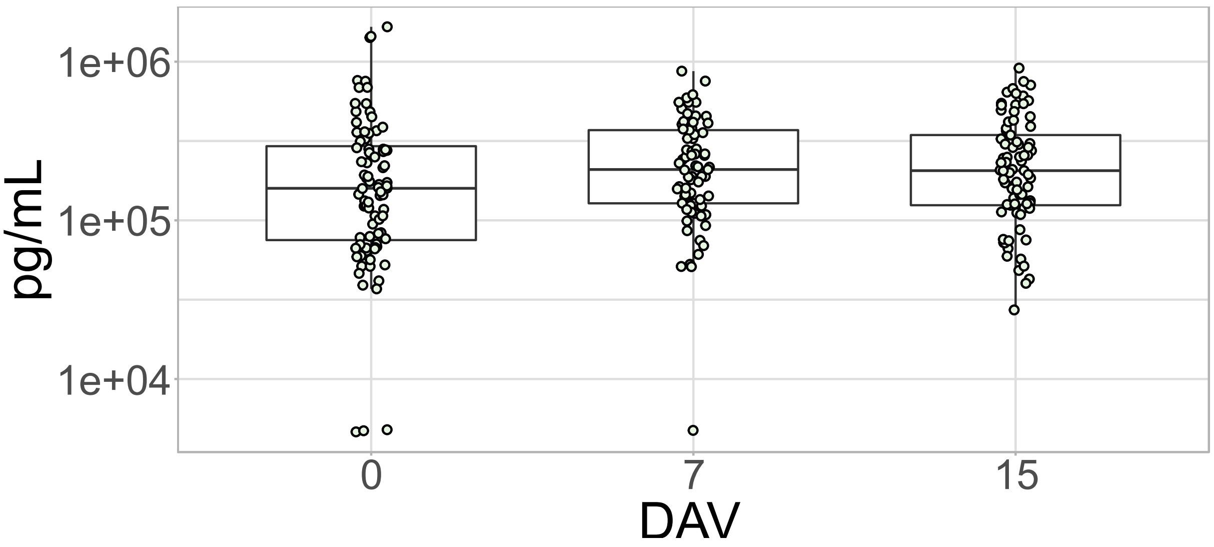

Supplementary Figure S2. Follow-up D-Dimer plasma quantitation after ChAdOx1 nCoV-19 vaccination analyzed at 0,7 and 15 DAV. Volunteers immunized with prime-boost protocols with ChAdOx1 nCoV-19 on days 0 and 90 respectively. Data represented in boxes with mean and interquartile interval of D-Dimer plasma quantitation in pg/mL. Comparisons between timepoints using Kruskal-Wallis with Dunn's multiple comparison post-test. Sample number varying according to follow-up segment loss. DAV = Days After Vaccination.

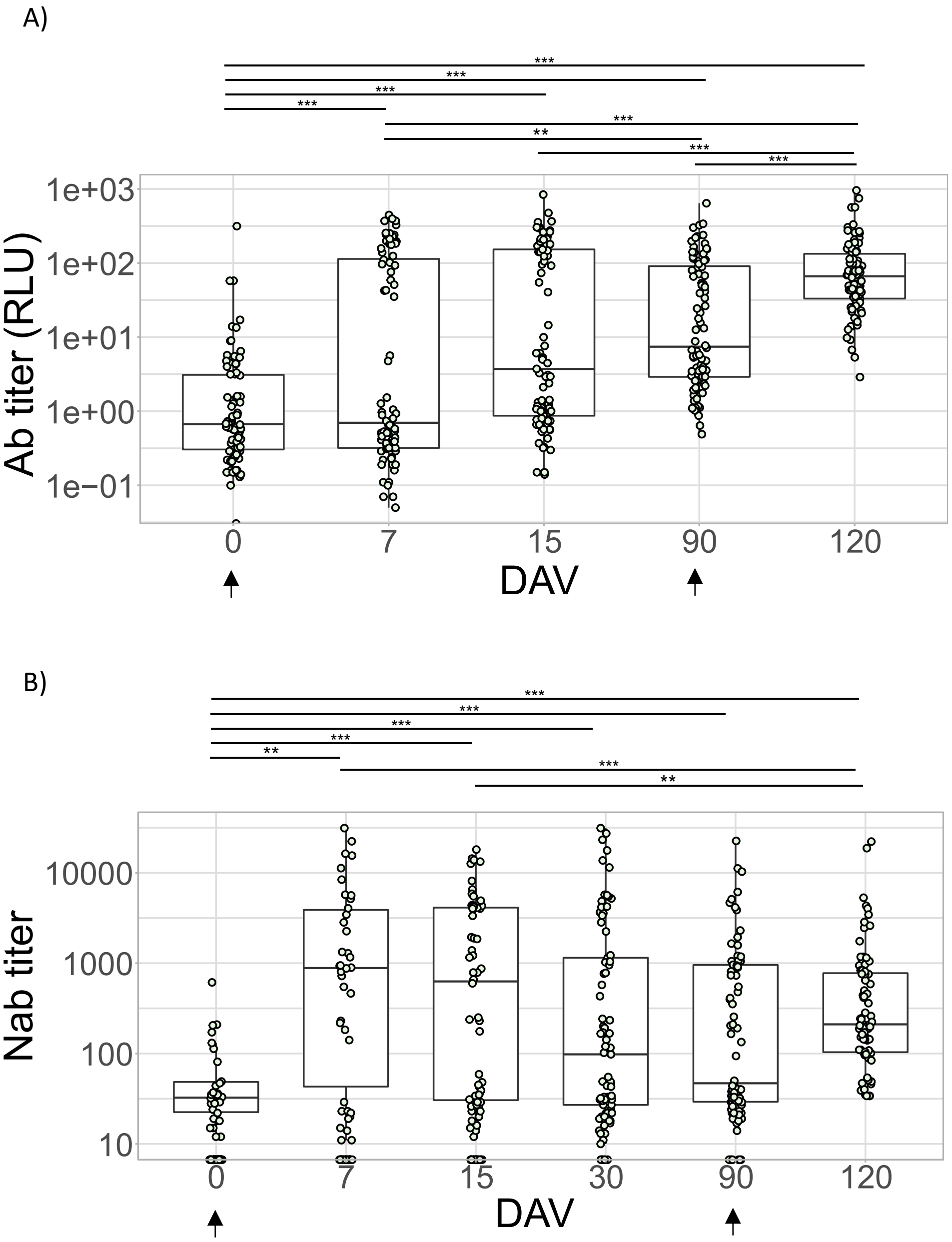

Supplementary Figure S3. Follow-up humoral analysis after ChAdOx1 nCoV-19 vaccination analyzed at 0,7, 15, 90 and 120 DAV. Volunteers immunized with prime-boost protocols with ChAdOx1 nCoV-19 on days 0 and 90 respectively (indicated by arrows). Data represented in boxes with mean and interquartile interval of (A) total IgG anti-RBD and (B) Nabs. Total IgG anti-RBD (RBD region-Spike protein of SARS-CoV-2 an ACE-2 receptor ligand) ELISA expressed by RLU (Relative Light Units). Nabs titer quantified by $PRNT_{50\%}$. Comparisons between timepoints using Kruskal-Wallis with Dunn's multiple comparison post-test. Considering *p<0.05, **p<0.01, and ***p<0.001. Sample number varying according to follow-up segment loss. DAV = Days After Vaccination. Nabs=Neutralizing antibodies.

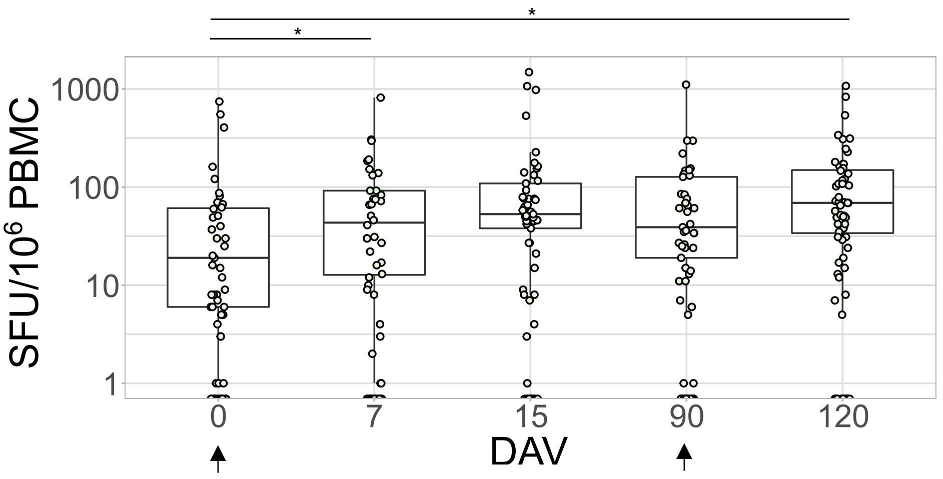

Supplementary Figure S4. Follow-up IFN-γ secretion quantitation after ChAdOx1 nCoV-19 vaccination analyzed at 0,7, 15, 90 and 120 DAV. Volunteers immunized with prime-boost protocols with ChAdOx1 nCoV-19 on days 0 and 90 respectively (indicated by arrows). Data represented in boxes with mean and interquartile interval of D-Dimer plasma quantitation in pg/mL. Comparisons between timepoints using Kruskal-Wallis with Dunn's multiple comparison post-test. Considering *$p<0.05$, **$p<0.01$, and ***$p<0.001$. Sample number varying according to follow-up segment loss. DAV = Days After Vaccination.
